# 3D-Printed Hermetic Alumina Housings

**DOI:** 10.3390/ma14010200

**Published:** 2021-01-03

**Authors:** Max Eickenscheidt, Michael Langenmair, Ahmad Dbouk, Dorit Nötzel, Thomas Hanemann, Thomas Stieglitz

**Affiliations:** 1 Laboratory for Biomedical Microtechnology, Department of Microsystems Engineering-IMTEK, University of Freiburg, 79110 Freiburg, Germany; michael.langenmair@imtek.de (M.L.); ahmad-dbouk@hotmail.com (A.D.); thomas.stieglitz@imtek.uni-freiburg.de (T.S.); 2 Institute for Applied Materials, Karlsruhe Institute of Technology, 76344 Karlsruhe, Germany; dorit.noetzel@kit.edu (D.N.); thomas.hanemann@kit.edu (T.H.); 3 Laboratory for Materials Process Technology, Department of Microsystems Engineering-IMTEK, University of Freiburg, 79110 Freiburg, Germany; 4 Bernstein Center Freiburg, University of Freiburg, 79104 Freiburg, Germany; 5 Brain-Links Brain-Tools, University of Freiburg, 79110 Freiburg, Germany

**Keywords:** ceramic, fused filament fabrication, alumina, scaffold, metal-ceramic bond, implant

## Abstract

Ceramics are repeatedly investigated as packaging materials because of their gas tightness, e.g., as hermetic implantable housing. Recent advances also make it possible to print the established aluminum oxide in a Fused Filament Fabrication process, creating new possibilities for manufacturing personalized devices with complex shapes. This study was able to achieve integration of channels with a diameter of 500 µm (pre-sintered) with a nozzle size of 250 µm (layer thickness 100 µm) and even closed hemispheres were printed without support structures. During sintering, the weight-bearing feedstock shrinks by 16.7%, resulting in a relative material density of 96.6%. The well-known challenges of the technology such as surface roughness (Ra = 15–20 µm) and integrated cavities remain. However, it could be shown that the hollow structures in bulk do not represent a mechanical weak point and that the material can be gas-tight (<10^−12^ mbar s^−1^). For verification, a volume-free helium leak test device was developed and validated. Finally, platinum coatings with high adhesion examined the functionalization of the ceramic. All the prerequisites for hermetic housings with integrated metal structures are given, with a new level of complexity of ceramic shapes available.

## 1. Introduction

Technical ceramics are preferred materials, especially in demanding use cases, such as aerospace components and medical implants. In these applications, ceramics are often used instead of polymers to achieve longevity in aggressive environments. Especially, ceramic-based circuit boards [1] or implant housings are of particular interest [2,3]. Both applications benefit not only from the wide temperature stability but also from the electrical properties and low permittivity for gases. For example, electrically insulated and hermetically sealed housings can be created without compromising the permeability of electromagnetic signals [4]. Furthermore, many alumina ceramics are considered biocompatible and biostable and have excellent electrical insulation properties [5]. In the manufacturing process, mostly cut or milled ceramics such as aluminum oxide, yttrium doped zirconium oxide, or zirconia-toughened alumina are used [2,3]. The ceramic plates are then subsequently functionalized at very high temperatures with screen printing pastes [4]. However, the subtractive manufacturing process of the substrates limits the possible housing shape, and the screen printing process is only possible on flat surfaces. Therefore, additive manufacturing (3D-printing) of ceramics can be used, which allows intrinsically complex shapes [6]. Printing implants would be a fast and effective way to produce personalized body replacements using computer-aided manufacturing. Exactly matched implants are commonplace in dental technology [7], but even here, milling technology is still the gold standard. Used as implanted package for electronics for customized replacement of a bone (part) would represent a further increase in structural biocompatibility and unobtrusiveness for the patient. In addition, greater freedom in circuit board design through exploring the third dimension [8] is also a promising technology for the future. Low-temperature cofired ceramics (LTCC) have been successfully employed for a number of applications in aerospace and biomedical devices [9], but the lamination technique and the high glass content limit the shaping and durability accordingly. The printing of a ceramic with high relative density could offer more possibilities.

Additive processes have been intensively studied in the last years [6,10]. By slurry-based technologies like Vat Polymerization or Inkjet Printing, very fine detailed structures can be realized but the devices are very expensive. Due to crosslinked photopolymers, Vat Polymerization needs very long debinding times [11], but it reaches relative densities > 99% [12]. For Inkjet Printing due to small nozzle sizes, submicron powders are essentially [6]. Powder-based technologies like Direct Laser Sintering or Binder Jetting leads usually to highly porous parts [10]. Extrusion based technologies like Fused Filament Fabrication (FFF), also known as Fused deposition Modelling (FDM), or Robocasting, use inexpensive devices. While Robocastings post-processing times due to low binder content are short, FFF allows more freedom of design because of mechanical resistant green bodies [6]. The main drawback of FFF is the method of inherent voids between the lines that could result in a low density of sintered parts. Nevertheless, it is feasible to achieve densities >99% [13].

The study’s objective is the feasibility of the new materials (filament) in a combination with the FFF-printing technique for special applications. For an envisioned long-term use of the printed material as an implantable device, the mechanical stability and post-processing must be examined in addition to the structural resolution on the fired workpiece. The envisioned applications are based on the packaging of electronics in a hermetically sealed housing. Therefore, the gas-tightness of the material itself will be investigated by helium leak testing, for which a volume-free helium leak tester was developed and used. The special tooling created for the commercially available helium leak tester is based on published studies, but additional customization was integrated to feature even lower helium pollution within the test device. In a second step, the functionalization of the ceramic parts with, e.g., integrated electrical conductive traces and solder joints, is realized with a low-temperature sputtering process of platinum. An additional thin tungsten/titanium adhesion layer ensures a strong bond beyond mechanical interlocking [14] of the metallization towards high durability of the system. According to the prototyping process of the FFF, a laser ablation process is chosen for structuring the metallization. The combined process for local metallization is possible on the complex topography of the ceramic, thus providing more opportunities for functionalized devices with integrated sensors [15], reactors, catalysts, or mixers [16]. At last, the advantages of the technology are proven by printing two sophisticated three-dimensional shapes without supporting structures. Examples with large overhangs and integrated channels were chosen, ceramic structures that have not yet been reported using the FFF method.

## 2. Materials and Methods

### 2.1. Ceramic Printing with Fused Filament Fabrication

Ceramic samples were prepared with the FFF, using tailor-made raw materials. The material compositions and print parameters have been published elsewhere (feedstock Polyethylene/Paraffin (PE/PW) [13] and feedstock Polyethyleneglycol/Polyvinylbutyral (PEG/PVB) [17]) and are described only with the most important steps. The green alumina filaments consisted of ceramic filler particles with 0.1 µm size (TM-DAR, Tamai Chemicals, Tokyo, Japan) in a concentration of 50 vol% (equals 81 wt.% [13] and 77.5 wt.% [17] respectively). The filament was prepared in a one screw Extruder (Noztek pro, Noztek, Shoreham, UK) at temperatures of 100–130 °C with a diameter of approximately 3 mm.

All designs were created using SolidWorks 2014 (Dassault Systèmes. Vélizy-Villacoublay, France) and sliced using Cura (Ultimaker, Geldermalsen, The Netherlands) software. Printing the ceramic components was performed using a modified desktop fused filament fabrication (FFF) printer (X350 pro, German RepRap, Feldkirchen, Germany).

The nozzle head sizes were 0.4 and 0.25 mm resulting in 200 µm and 100 µm layer thickness, respectively. The smaller nozzle size was used with the PEG/PVB feedstock with which only the implant housing was printed here (see Section 3.3). The speed of the printer head was set to be 20 mm·s^−1^ with a filling of 110%. A cooling fan was used to cool down the printed layer before the deposition of the second layer on top, while the bed sheet, covered with an adhesive tape, was held constant at 60 °C. The individual layers were applied in adjacent tracks, which were rotated alternately by 90° from one layer to another.

The post-processing was performed in two different steps, debinding (chemically and thermally) followed by sintering. First, paraffin was dissolved in n-hexane at 50 °C and polyethyleneglycol in distilled water at ambient temperature for 24 h. The solvent debinding removes one binder composition and opens pores inside the sample where the Low-Density Polyethylene (LDPE) or rather Polyvinyl Butyral (PVB) stayed to preserve the shape of the printed green ceramic. Followed by a stepwise thermal debinding process up to 500 °C [17] to remove most of the remaining polymer left inside the sample. The final sintering process was performed at 1400 °C with a heating rate of 3 K·min^−1^ and a holding time of 6 h.

As reference systems, two different other aluminum oxide plates are used. we chose substrates of 96% Al_2_O_3_ (Rubalit 708 S, CeramTec, Marktredwitz, Germany), which were cut to 0.635 mm thick plates by laser cutting process (A.L.L. Laser Technik GmbH, Munich, Germany) and less crystalline High-Temperature Co-Fired Ceramics (HTCC 44000) supplied by ESL Europe (Reading, UK) [18]. Four layers of HTCC (thickness of 200 µm), each rotated by 90° to the previous layer, were laminated with pressure and elevated temperature. During sintering, the plate shrinks by 16.7% resulting in an overall thickness of 0.666 mm.

The density of the fired ceramics was determined by weighing the compounds in air and water at 22 °C (BP 211 D, Sartorius AG, Göttingen, Niedersachsen, Germany). The difference in weight caused by the displaced fluid (Archimedes’ principle) is proportional to the density of the body, knowing the density of the fluid.

### 2.2. Surface Preparation, Metal Deposition and Assembly

Smoothening of the ceramic surfaces was performed utilizing a two steps mechanical grinding scheme with a grain size of 82 μm and 46 μm (MD-Piano series disks by Struers GmbH, Willich, Germany). During the manual grinding process, distilled water was used as an intermediate medium between the disk and the sample. Afterward, the surfaces were cleaned by immersion in a water-based alumina cleaning solution with a basic pH solution [12] for 10 min on a rocking plate. Succeeding immersion in isopropanol solution with sonification for 10 min further removed dust and other foreign particles on the surface. Finally, the samples were washed in DI water and dried using an air gun.

The ceramics were functionalized by a structured metal film on the cleaned ceramic surfaces. For this purpose, the samples were coated with a metal layer stack of tungsten/titanium (WTi10) and platinum (50 nm and 500 nm respectively) by sputter deposition (Leybold Univex 500, Leybold Vacuum GmbH, Cologne, Germany). WTi10 acts as an adhesion promoter to ensure a higher adhesion of the sputter-deposited platinum [19] which is used as the conductor in active implants. The structuring of the metals was realized by masking the uncoated parts with polyimide adhesive tape before deposition and by laser ablation with an infrared Nd:YAG nanosecond laser (ACL Laser GmbH, Nohra, Germany) at a wavelength of 1064 nm after deposition and peeling off of the coarse adhesive tape mask. Soldering was done manually with a soldering iron at 350 °C using a lead-based solder (Sn62Pb36Ag2, Felder Lottechnik, Oberhausen, Germany).

### 2.3. Testing Procedures

Lateral destructive forces were studied with the bondtester Dage-4000 series PXY (Cartridge: DS100KG, Nordson Dage, Westlake Ohio, OH, USA). The method allows the evaluation of the point of failure between the two attached materials, in which the ultimate shear strength can be calculated by dividing the force over the cross-sectional area of shear. The chisel can achieve a maximum horizontal pushing force of 88.2 N. The same machine was used to perform pull tests on solder joints between sputtered metal structures on ceramics and soldered copper wires (Cartridge: WP10KG) where the metalized ceramic was fixated and the test tool pulled on the soldered wires (which were soldered perpendicular to the ceramics).

The roughness of the printed and sintered alumina surfaces was measured using a confocal microscope LSM 5 Pascal (Carl Zeiss, Oberkochen, Germany) with a laser wavelength of 488 nm. The presented surface roughness is the mean height, whereas the lowest measured value is set as zero. The mean peak to valley value is the averaged value of the measured extrema.

The gas permeability of flat materials was tested with a customized clamping device for planar samples, mounted on a calibrated commercial helium fine leak tester (SmartTest HT570, Pfeiffer Vacuum Technologies, Asslar, Germany). Two sealing rings (5.3 and 7.0 mm inner diameter), defining the exposed areas of the top and bottom side were made of nitrile material (NBR), purchased from Esska GmbH (Hamburg, Germany). The minimum wall thickness of the attachment, which is under vacuum, is 1 mm and the helium feeds are 3 mm in diameter. The whole attachment was made by turning stainless steel.

## 3. Results and Discussion

### 3.1. Bulk Structures and Geometric Properties

Plate-like planar structures using the PE/PW feedstock were printed to examine the general surface properties of the sintered devices. The binder component LDPE ensured the stability of the green body. After the binder was removed with n-hexane, the green body is still dimensionally stable and transportable. In the subsequent sintering process, the technical ceramics are brought into a firm bond. It turned out, that the thermal debinding process [13,17] is a crucial process step to prevent crack formation during sintering and to ensure structural integrity and gas tightness.

**Figure 1:**
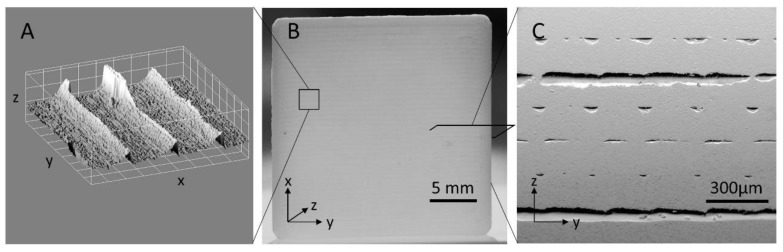
Plate sample: (**A**) Surface topography measured by a confocal microscope (grid pattern 100 µm) reveals a high roughness of a printed plate (**B**). The transmitting light shows homogeneous bulk, but also the surface line structure. (**C**) SEM images of a grinded cross-section of a plate fabricated using fused filament fabrication (FFF) reveals internal cavities due to the printing process and viscosity of the heated filament.

The printed forms had a consistent volume without cracks or macroscopic defects (Figure 1B). However, images of the cross-sections and surfaces revealed the process-related internal voids and roughness that reflect the shape and viscous properties of the extruded filament of the printing process (Figure 1A,C). The change of the printing direction by 90° from one layer to the next becomes visible through the alternating cavities parallel and into the section planes (Figure 1C). Note, that during all debinding and sintering steps no mechanical force was applied to the samples, which allows complex designs with hollow structures. This fact, in combination with the mechanical stability of the green bodies before firing, facilitated the reliable and reproducible production of complex geometries in the fired alumina workpieces.

Test samples printed with a nozzle diameter of 250 µm and a layer thickness of 100 µm showed linear shrinkages of 20.4% and relative densities of 99.4% for feedstock PE/PW and shrinkages of 20.75% and relative densities of 97.1% for feedstock PEG/PVB [13,17,20]. The density of the here printed plates with a nozzle diameter of 400 µm and layer thickness of 200 µm results in a lower relative density of 96.6%, which is in the same range as the used commercial Rubalit 708 S (density 96%). Orlsovská et al. [21] showed increasing voids/space between adjacent lines with increasing layer thickness, which is helpful for debinding but could decrease mechanical properties.

The density of the printed and fired ceramic with the voids was determined by the Archimedes method (water at room temperature) to 3.84 g·cm^−3^ (relative density 96.6%). In comparison, commercially available alumina (96%) and thermo-compressed HTCC 44,000 had densities of 3.8 g·cm^−3^ and 3.7 g·cm^−3^, respectively. The high proportion of Al_2_O_3_ in the feedstock resulted in very dense bulk, unaffected by the holes and voids in the printed ceramic. An appropriated extension would be hot isostatic pressing to heal the defects and to gain finer grain sizes while preserving the intended shape [22]. The subsequent filling of the cavities is another option and has already been considered for dental implants [23].

On all sides of a printed plate, the grooves and traces of the printing process can be seen on the surface of the ceramic (Figure 1A). It must be noted, that the substructure can also be seen on the bottom side, representing the first layer deposited on a base plate (Figure S1). For an exact quantification, the surface roughness was determined (Table 1) by scanning the surface with a confocal microscope in a representative 900 × 900 µm^2^ area. The mean derivation of the surface height related to the mean surface level and the maximum height differences measured show a 20–30 times higher surface roughness as commercially available laser cut ceramic plates. The most dominant profile topography is clearly given by the printing path of the deposited filament. The surfaces can be grinded in order to achieve a surface roughness comparable to commercial alumina plates. Optical control during grinding is mandatory since the next unevenness is expected after about 150 µm due to the embedded cavities during the printing procedure. It may be possible to achieve even lower roughness with finer abrasives, but the grain- and void-structure of the bulk will define a lower limit. The voids could be reduced or possibly avoided by changes in the printing parameters [24], but the initial composition of the material has proven to be extremely beneficial for printing complex 3D structures.

### 3.2. Mechanical Properties

**Figure 2:**
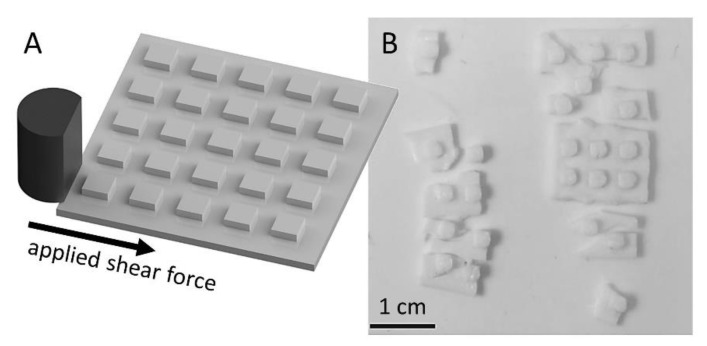
(**A**) Printed squares with an edge length of 2.5 mm and a height of 1 mm arranged on a ground plate of 3 × 3 cm^2^ and a height of 1 mm were sheared off and the force until breakage was measured. (**B**) In all cases, the 1 mm thick carrier plate was broken, if a breakage had occurred. In six cases, neither the base plate nor the squares with a maximum of 300 N could be destroyed.

The 3D-printing procedure with subsequent sintering produces a layered structure with patterns of cavities, which could strongly influence the integrity of the bulk. Therefore, the mechanical properties of the critical plane between different printing layers were tested with shear tests instead of performing a three-point breaking test of a full plate. Test plates were printed with freestanding squares with an area of 6.25 mm^2^ and a height of 1 mm. These squares were then loaded horizontally with the chisel of the bond tester (Figure 2A). In case the sample was not sheared off in one direction, the sample was rotated 90° and the same test again was performed. An HTCC layered structure with the same dimensions but structured with a subtractive process (nanosecond-laser) served as a reference. These samples show a high mean resistance and were torn off at 275 ± 18 N (*n* = 5), whereby fracture patterns occurred exclusively in the base plate. The printed samples showed a slightly lower breaking strength at 257 ± 79 N. Optical control showed that the individual squares were not be sheared off in any case, but that the base plate was broken in all cases (Figure 2B). In six cases of testing the printed and sintered pillars, no shearing or breaking of the base plate was achieved during the measurement with a maximum force of 300 N. Indicating, that the weakness of the printed ceramic is not in the laminar structure of the printing process but in defects within the bulk. Concluding, a mean shear force of at least 260 N, which must be applied to break off pieces from a 1 mm thick plate, is a sufficient value for most applications.

Calculating from the force until rupture to the ultimate shear strength of 41 MPa shows significantly lower values obtained from commercially available ceramics in the range of 130 GPa. However, the values are also not comparable, since in none of the cases shearing-off the cuboids worked. This indicates, that the layer-by-layer manufacturing process and the associated horizontal voids are not a direct weak point of mechanical integrity.

### 3.3. Hermeticity

**Figure 3:**
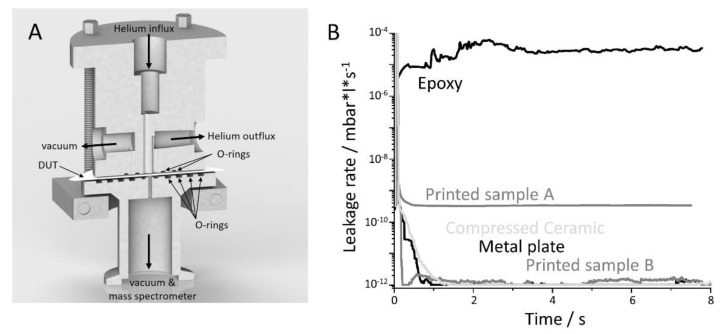
Helium leakage tests with the printed ceramics and positive as well as negative controls. (**A**) A volume-free helium leakage head to clamp a flat device under test (DUT) between different O-rings. Constant helium pressure of 1.1 bar is applied to the DUT from above and the mass spectrometer is connected on the opposite side. Removing the helium that diffuses through the inner top O-rings via a vacuum pump reduces cross-contamination. (**B**) The limit of helium flow is reached with metal plates and thermo-compressed ceramics. The thermo-compressed ceramic, as well as the printed ceramics, can also reach the limit or show a constant helium current smaller than the Gross-Leak Limit of 10^−4^ mbar·s^−1^, which is almost reached with an epoxy DUT.

Especially when the packaging of sensitive electronics is necessary, the 3D-printed cavities have to ensure a certain resistance to gas and fluid ingress. To quantify that parameter, we developed a volume-free chamber for a helium leakage tester (Figure 3A), in which various flat samples can be clamped. A defined helium atmosphere can be applied to one side of the sample and the mass spectrometer of the helium fine leakage tester is connected to the other side. The top side O-rings are 5.3 mm inner radius and a second 10 mm ring closes a gap where excess helium leaking through the first O-ring is transported away from the sample. This approach reduces cross-contamination via the rubber seals and via leakage from the ambient air. Nitrile rubber (NBR) O-rings were used since it exhibits the lowest permeability to helium gas (12.8 × 10^−12^ cm^2^·s^−1^·mbar^−1^) in a steady-state compared to Neoprene (16.8 × 10^−12^ cm^2^·s^−1^·atm^−1^) or Viton (38.5 × 10^−12^ cm^2^·s^−1^·atm^−1^) [25]. The observed area on the detector side can be varied with different O-rings with an inner radius of 2, 7, 12, 17, and 21.7 mm respectively. In the present study, a radius of 7 mm was used, which was slightly larger than the area exposed to helium on the top of the flat sample. The mounting bracket is screwed tightly, thus minimizing the vibration of the Helium Fine Tester affecting the sealing of the O-rings. The mass spectrometer measured the amount of helium diffusing through the device under test (DUT) with a minimum detection threshold of 10^−12^ mbar·s^−1^. Different printed ceramics were compared with a 1 mm thick grade 301L stainless steel as positive control and a 500 µm thick epoxy sheet (Epotek 301-2) as a negative control.

**Figure 4:**
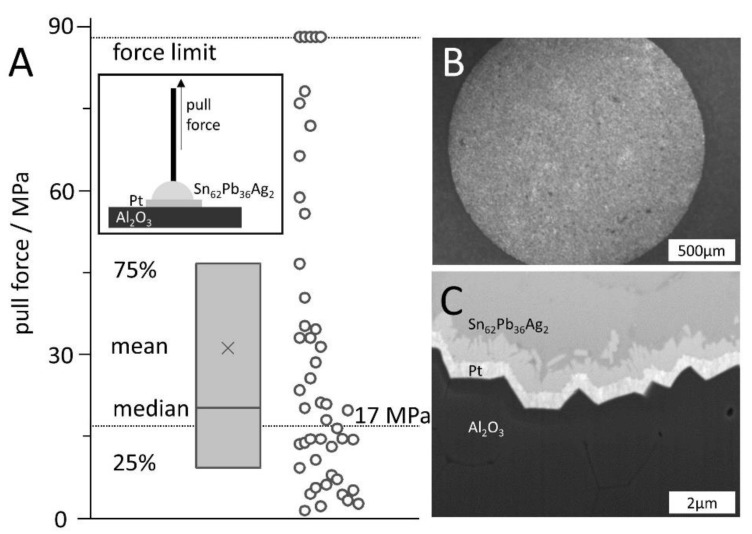
Pull test on FFF-printed ceramic-metal-pin sequences (see insert). (**A**) Pull force to destructive disjoin the pin and the ceramic. Box plot indicates 25–75% interval, the star marks the mean value, whereas the centerline is the median value. (**B**) Photo of a laser structured solder pad on aluminum oxide ceramics. (**C**) Electron microscope image of the cross-section of the layered composite of aluminum oxide ceramics (bottom), WTi10/platinum (500 nm), and the solder material (top). Between platinum and solder tin an intermetallic transition of about 0.75 µm was formed.

The leakage rates of different materials were measured up to 8 min while exposed to helium (Figure 4B). In the volume-free measuring device, the upper side of the devices is exposed to a constant 1.1 bar helium pressure and the lower side to a helium leak tester with a stable 10^−8^ mbar vacuum. The epoxy material Epotek, used for chip under-fills, reached almost the limit of the gross leak of 10^−4^ mbar·s^−1^ and showed the expected constant flux of Helium through the material [26]. Leakage rates below the detection limit were measured with a stainless steel plate for chamber validation purposes. This behavior was expected since the material is also used to build high vacuum systems. It proves that the rubber seals and the suction concept on the top were very effective since even after 10 min no cross-contamination of helium was detectable in the measuring chamber. The penetration of one polymeric O-ring was estimated to take about 3 min [25]. In literature, leakage experiments in the range of a few minutes are described to a large extent [4,25,27]. A long-term investigation on the permeability of the materials may also be desirable, including more complex equipment and sealing techniques [28].

In the presented experiment two 1 mm thick printed and sintered alumina plates were successfully measured yielding constant and reliable leakage values. One of the printed ceramics showed values below the detection limit (Figure 3, Printed sample B) of the mass spectrometer, whereas the second device (Printed sample A) showed a constant leakage rate of 3.3 × 10^−10^ mbar·s^−1^. Consequentially, tight ceramic can be produced with the presented FFF technology, but the reproducibility has to be further optimized. The 0.6 mm thermo-compressed HTCC with an Al_2_O_3_ content of 96% achieved a comparable good result, with a leakage rate near the detection limit of the tester (Figure 3, Compressed ceramic). The results are consistent with other studies, showing, that high amounts of alumina result in “gas tight” packages. Thermo-compressed alumina in particular often shows a large number of microcavities [29], but these do not have a particularly negative effect on the barrier properties, as well as the presented printed ceramics. According to the military standard MIL-STD-883, a leak rate of 2.36 × 10^−9^ mbar·s^−1^ is considered to be gas-tight, which was successfully reached by both printed and sintered samples. In the case of hermetic packaging, however, the amount of enclosed volume and the envisioned service life of the capsule must also be taken into account [30]. Therefore, the leakage rates measured may not be sufficient for devices that have a too high surface/volume ratio. On one hand, the lifetime can be improved by desiccants, for example, which keeps the air humidity content low for a longer period of time [31]. On the other hand, the Al_2_O_3_ precursor can be mixed with other technical ceramics like ZrO_2_ to lower the permeability [32] or impregnate with nano-sized particles.

In order to ensure adequate packaging, the ceramic could also be coated with a thin layer of metal to achieve an even lower permeability to gases and moisture [33]. In the same way, structured metal layers lead to functionalization of the surfaces and to the realization of conductive traces, solder pads for electronics, the integration of sensors [15], the construction of reactors [34], catalysts, or mixers [16].

### 3.4. Surface Metallization

For all envisioned fields of application, metallization of the ceramics for conductive tracks or sensor systems is advantageous when good bonding between the coating and the base ceramic is ensured. The strength of the bonds between the aluminum oxide and the metallization (a platinum layer) was evaluated by recording the maximum force to failure in pull experiments (Figure 4). For this purpose, the WTi10 (50 nm)/Platinum (500 nm) layer sequence was deposited on polished and cleaned surfaces of the FFF-printed ceramics and then laser structured by laser ablation to create defined round pad geometries with 1.9 mm as well as 1.4 mm diameter (Figure 4B). A pre-tinned 1 mm thick and 2 cm long copper wire was attached by soldering. The mean ultimate tensile strength needed to de-attach the wires was 31.1 MPa (*n* = 47). Also, the median strength (20.1 MPa, *n* = 47) was higher than the conservative threshold of 17 MPa, set by a comparable study showing also a 32 MPa mean adhesion strength on commercial alumina [19]. No clear difference in maximum pull force between the copper rods soldered on the two different pad geometries was found. The most common failure modes were a (partial) de-attachment of the metallic film from the aluminum oxide and a break at the intermetallic phase, as revealed by optical inspection.

A cross-section of the layer stack was produced by focused ion beam cutting (Figure 4C) and reveals a homogeneous and conformal deposition of the two metal layers. In addition, an intermetallic phase between the platinum finish and the soft solder was found. Furthermore, small cracks in the bulk material of the alumina are visible.

In contrast to other established processes for structuring metals on ceramics, especially with screen printing techniques, no plane samples and no high-temperature steps during deposition are required. Challenges due to coefficient of thermal expansion mismatches or pressure distributions during high-temperature firing on the shape of the ceramic to be metalized are minimized. Likewise, liquidus diffusion should be negligible, which occurs for medical implants with platinum-gold metallization embedded in a glass matrix [19]. In contrast, the developed sputter-deposited WTi-Pt metallization showed excellent long-term stability on smooth Al_2_O_3_ at elevated temperatures in air and saline [19]. The adhesion promoter WTi10 increases the adhesion strength by a factor of three [14], presumably forming a covalent bond with the oxygen atom of the ceramic substrate [35]. There are hardly any restrictions on the materials that can be sputtered or coated with electrodeposition, e.g., palladium, gold, or iridium oxide.

The fracture is partly cohesive and partly adhesive in nature and shows extended scattering from 2 MPa up to the detection limit of 88 MPa. Unlike the previous studies [14,19], partial adhesive failure of the metal-ceramic interface was observed here, which may explain the lower adhesion and large variance. One difference was the larger surface roughness, which is decisive for the wettability during sputter deposition as well as the soldering process [36]. In addition, roughness would also improve mechanical interlocking. However, it was observed, that stable connections are difficult to achieve when the surface topology is too large like with pristine printed devices (Figure 1A). Another difference is the presence of little cracks in the uppermost surface of the Al_2_O_3_ adhesive failure, which probably leads to adhesion failure and lower strength.

The spatial resolution of the near-infrared laser of about 30 µm is sufficient and comparable to commercial PCB processes. The use of ultraviolet lasers would allow the reduction of distances between metal tracks of less than 20 µm [37]. Sputtering and laser ablation are possible on the complex topography and support of the rapid prototyping approach.

### 3.5. Realization of Complex Structures

**Figure 5:**
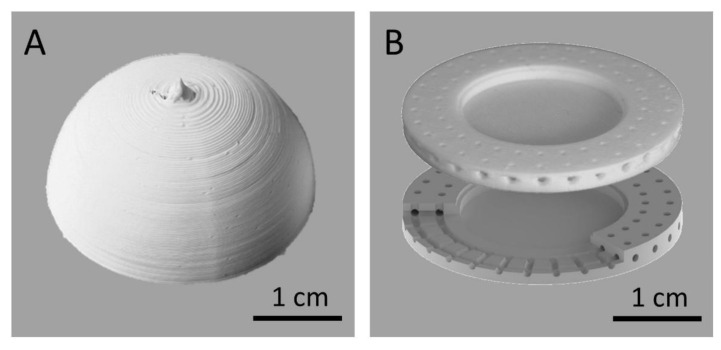
Two successfully printed three-dimensional structures with pronounced overhangs. (**a**) a half-sphere with a wall thickness of three lines is shown, which has an outer diameter of 3 cm. (**b**) implant housing with an inner cavity and surrounding embedded channels with a diameter of 1 mm (Top: 3D-printed ceramic after sintering; Bottom: drawing).

For illustrative purposes, several complex structures with overhangs have been realized, which highlight the unique value of FFF printing (Figure 5). On the one hand, completely free-standing hemispheres were printed with a wall thickness of 3 filament perimeters (corresponding to 2 mm before and 0.8 mm after sintering). The dome experiences hardly any distortion during the printing process and the viscosity of the filament allows a dimensionally stable deposition at an inner radius of 30 mm. After sintering, a lateral shrinkage of 20–23% did still lead to an intact and closed hemisphere with 30 mm diameter, although some surface defects are visible on the pole (Figure 5a), due to insufficient heat removal of the cooling fan. The uneven surface structures (staircases) are a characteristic risk of the FFF technique. A second application involves embedded channels with a (pre-sintered) radius of only 500 µm, which can be transferred to sintered devices that are true to shape in all directions. Such channels can be used for chemical reactors, grow chambers, sensor systems, or scaffolds for implants. The advantage of the additive approach compared to subtractive processes becomes apparent, which can never yield to embedded structures. Using the channels for microfluidic devices, the flow characteristics of the channels must be further investigated, as the rough inner walls certainly have an influence. The specific prototype, shown in Figure 5b was designed as implant housing with integrated electronics [4,38] in the center. The canals were placed at the edge of the device for direct osseointegration [39,40], that attached bones can grow into them and form a firm mechanical bond. It is the first time that such complex 3-dimensional structures are realized in highly pure alumina ceramic with unprecedented freedom in design. It is notable, that for all shown overhangs and embedded channels no supporting structures or coatings on printed polymers were needed [41].

## 4. Conclusions

For the first time, it could be shown that an FFF process with alumina as a solid component, dispersed in a polymer-based binder, can be used to print the smallest overhangs and cavities that cannot be achieved in subtractive processes. The complex 3-dimensional structures are also stable in the as-deposited state and keep their shape during sintering (with the expected shrink). Staircases and voids are still a challenge of the technology, but structures on the scale of the nozzle size can be realized, which is rather unusual for filament-based additive manufacturing technologies. Also, the surface roughness is a known drawback of the process, measured to 150 µm. Critical areas can be easily polished to come close to the surface roughness of commercially available semi-finished goods.

Mechanical shear tests showed that the process related voids within the bulk material has hardly any effect on the mechanical stability. The connection between the printed layers was never the weakest point in the applied testing procedure. The failure forces were slightly lower than the negative manufactured ceramics and could be increased by minimizing the voids as the weakest point in the bulk. Hermeticity of the printed ceramics was successfully tested by a customized device for gas permeability measurements. The printed ceramics have a leakage rate that is comparable to compressed ceramics and metal plates in the range of measuring accuracy, which makes them suitable candidates for packages in active implantable medical devices like neural implants. Other material properties, such as temperature resistance, heat spreading, and low electromagnetic shielding, should be preserved and thus an application in space applications is also of interest.

Subsequent functionalization, especially metal depositions, and structuring can also be easily performed. The bonding force of the printed surface is comparable to commercially manufactured ceramics. Since active implantable medical devices come with a silicone rubber encapsulation (polydimethylsiloxane-PDMS) around the ceramic package (e.g., to improve structural biocompatibility and to provide insulation of wires [42], alumina with 96 wt% Al_2_O_3_ is preferred. Especially for the intended applications in the medical or transport sector, the densities of the material achieved here are better than the used commercial one and the process shows great potential.

## Figures and Tables

**Table 1:** Surface roughness was measured as arithmetic mean deviation of each sampling point on one surface of a sample (Ra) or as the maximum detected peak-valley value on the top and bottom surfaces (Rz) of the printed ceramics before and after grinding.

Sample		Printed	Grinded	Commercial Plate
Ra/µm	bottom	15.1 ± 4.0 (*n* = 19)	1.24 ± 0.21 (*n* = 9)	0.66 ± 0.08 (*n* = 6)
	top	20.9 ± 7.3 (*n* = 15)	1.23 ± 0.47 (*n* = 7)	
Rz/µm	bottom	152.1 ± 20.1	13.04 ± 1.72	8.18 ± 2.91

## Data Availability

The data presented in this study are available on request from the corresponding author.

## Supplementary Materials

The following are available online at https://www.mdpi.com/1996-1944/14/1/200/s1, Figure S1: Surface roughness of a printed ceramic plate measured by confocal microscopy. (a) Bottom and (b) top side of a pristine printed sample after sintering. (c) Bottom and (d) top side after grinding of the same sample.
