# Supplementary material for: 3D-Printed Hermetic Alumina Housings
Source: Materials (Basel). 2021 Jan 3;14(1):200. doi: 10.3390/ma14010200 (PMC7795362; doi:10.3390/ma14010200)
Supplement: Supplementary file 1 [file materials-14-00200-s001.pdf]

materials

Supplementary

3D-Printed Hermetic Alumina Housings

Max Eickenscheidt [1,*], Michael Langenmair [1], Ahmad Dbouk [1], Dorit Nötzel [2], Thomas Hanemann [2,3] and Thomas Stieglitz [1,4,5]

[1] Laboratory for Biomedical Microtechnology, Department of Microsystems Engineering-IMTEK, University of Freiburg, 79110 Freiburg, Germany; michael.langenmair@imtek.de (M.L.); ahmad-dbouk@hotmail.com (A.D.); thomas.stieglitz@imtek.uni-freiburg.de (T.S.)
[2] Institute for Applied Materials, Karlsruhe Institute of Technology, Karlsruhe, Germany; dorit.noetzel@kit.edu (D.N.); thomas.hanemann@kit.edu (T.H.)
[3] Laboratory for Materials Process Technology, Department of Microsystems Engineering-IMTEK, University of Freiburg, 79110 Freiburg, Germany
[4] Bernstein Center Freiburg, University of Freiburg, 79104 Freiburg, Germany
[5] Brain-Links Brain-Tools, University of Freiburg, 79110 Freiburg, Germany
* Correspondence: eickenscheidt@imtek.de; Tel.: +49-761-2036-7636

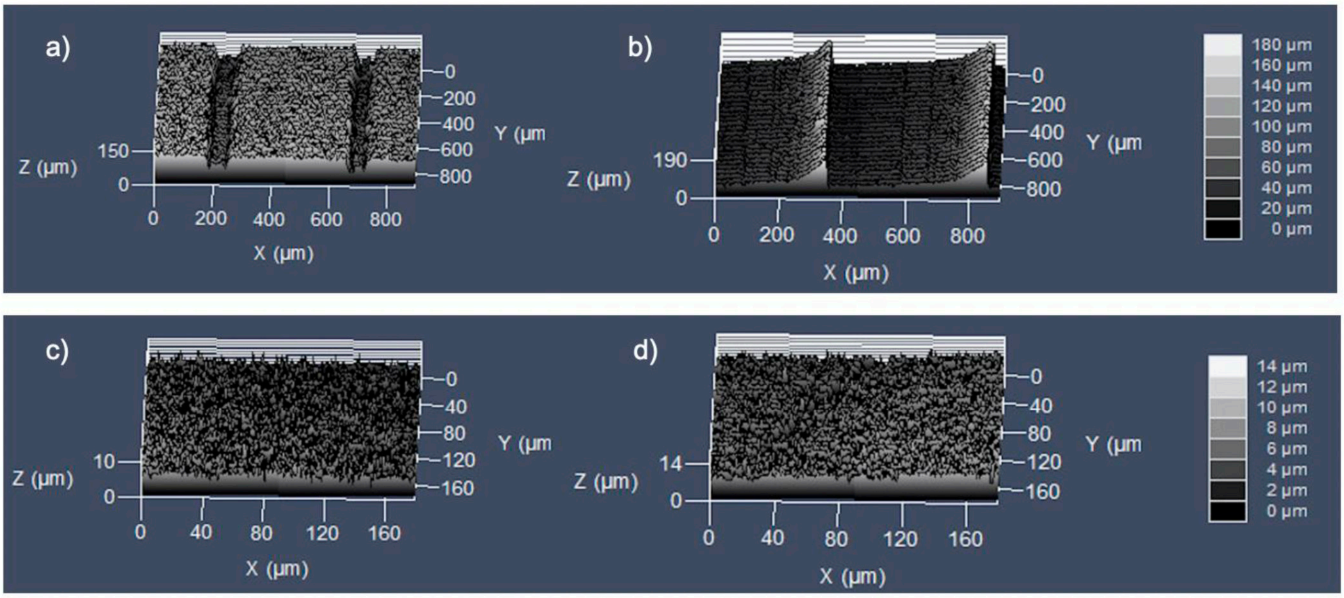

Figure S1. Surface roughness of a printed ceramic plate measured by confocal microscopy. (**a**) Bottom and (**b**) top side of a pristine printed sample after sintering. (**c**) Bottom and (**d**) top side after grinding of the same sample.
